# Application of NGS Technology, Association Mapping, and Physical Mapping Technologies to Identify Candidate Genes Associated with Maize (*Zea mays* L.) Hybrid Yield

**DOI:** 10.3390/ijms27114847

**Published:** 2026-05-27

**Authors:** Jan Bocianowski, Agnieszka Tomkowiak, Ewelina Wagner, Daniel Lipiński

**Affiliations:** 1 Department of Mathematical and Statistical Methods, Faculty of Agriculture, Horticulture and Biotechnology, Poznań University of Life Sciences, Wojska Polskiego 28, 60-637 Poznań, Poland; 2 Department of Genetics and Plant Breeding, Faculty of Agriculture, Horticulture and Biotechnology, Poznań University of Life Sciences, Dojazd 11, 60-632 Poznań, Poland; agnieszka.tomkowiak@up.poznan.pl; 3 Plant Breeding Smolice, Group IHAR, Smolice 146, 63-740 Kobylin, Poland; wagner@hrsmolice.pl; 4 Department of Biochemistry and Biotechnology, Faculty of Agriculture, Horticulture and Biotechnology, Poznań University of Life Sciences, Dojazd 11, 60-632 Poznań, Poland; daniel.lipinski@up.poznan.pl

**Keywords:** maize, association mapping, molecular markers, yield, hybrids

## Abstract

Maize (*Zea mays* L.) is one of the most important cereal crops worldwide, with yield being a complex quantitative trait controlled by multiple genetic factors. The aim of this study was to identify molecular markers associated with maize yield using next-generation sequencing (NGS), association mapping, and physical mapping approaches. A total of 122 maize hybrids were evaluated under field conditions in a randomized complete block design with three replications. Phenotypic data were collected for grain yield, while genotypic data were obtained using DArTseq technology, resulting in the identification of 60,436 SilicoDArT and 32,178 SNP markers. After quality filtering, 25,078 markers were used for further analyses. Analysis of variance revealed statistically significant differences among hybrids in terms of yield (*p* < 0.001), with values ranging from 12.67 to 18.52 kg/10 m^2^. Genetic similarity among hybrids ranged from 0.434 to 0.957, indicating substantial genetic diversity. Cluster analyses based on phenotypic and genotypic data showed a lack of correspondence between yield performance and genetic similarity. Genome-wide association studies (GWAS) identified 2478 markers significantly associated with yield, including 47 highly significant markers (Logarithm of the Odds − LOD > 4.0). Individual markers explained between 2.4% and 18.7% of yield variation. Ten markers with the highest contribution to yield variability (13.30–18.70%) were selected as the most promising candidates for further breeding applications. These markers represent promising candidates for marker-assisted selection and genomic selection (GS) of high-yielding maize genotypes. These are some of the first positive results. The integration of phenotypic evaluation with high-throughput genotyping and association mapping provides valuable insights into the genetic architecture of yield and offers practical tools for the development of high-yielding maize cultivars.

## 1. Introduction

Maize (*Zea mays* L.) is recognized as one of the most important and oldest cultivated plant species. It is generally accepted that the centers of origin of maize are Mexico and Central America, where it has been cultivated for approximately 4500 years. This species belongs to the tribe Maydeae within the family Poaceae [1,2,3]. One theory suggests that the most probable ancestor of maize is teosinte (*Zea mays* subsp. *parviglumis* H.H. Iltis and Doebley). Genetic loci such as *tb1* (teosinte branched 1) and *tga1* (teosinte glume architecture 1) played a crucial role in the transformation of teosinte into modern maize [4,5,6]. Currently, maize, together with wheat and rice, is among the most economically important cereal crops [7]. The cultivation range of maize is very broad, extending between 50° N and 40° S.

At present, maize grown for grain is cultivated on approximately 197 million hectares worldwide, making it the second most economically important crop after wheat. For comparison, wheat occupies 216 million hectares, and rice 165 million hectares [8]. Annual global maize grain production currently reaches approximately 1137 million tonnes, significantly exceeding that of rice and wheat. Over the last 25 years, maize production has more than doubled, driven by both substantial yield increases and expansion into new cultivation areas [8]. Among the three major cereals, maize yields have increased by nearly 2 tonnes per hectare (from 3.9 to 5.8 t/ha) over this period [9].

The aforementioned intensive increase in maize yields would not have been possible without biological progress. This progress can be defined as an ecological intensification of agricultural production, based on the genetic improvement of plants, enabling them to utilize natural resources and industrial inputs more efficiently [10]. These improved plants also exhibit superior quality in terms of human requirements, ultimately reducing production costs. The development of biological progress in various crop species is undoubtedly influenced by the introduction of new genetic variability [11]. The search for new genes of economic importance remains a key objective in modern plant breeding.

Currently, it is assumed that breeding priorities, including those in maize breeding, involve the introduction into production of varieties with higher utility value, i.e., increased yield potential and improved nutritional, feed, and technological quality of the harvested product. It is also essential to enhance plant resistance to both biotic and abiotic stresses [12]. Modern maize breeding worldwide relies on a wide range of molecular genetics techniques, primarily applied in two main areas. The first concerns decision-making in selection based on DNA nucleotide sequence analysis, while the second focuses on expanding genetic variability in breeding populations through genetic modifications. These modifications primarily involve the creation of plant organisms containing genes derived from other species [13,14]. This not only provides attractive prospects for achieving biological progress but also opens new possibilities for the utilization of maize as well as other crop species [15].

The DArT platform provides analyses based on NGS-DArTseq technology [16]. The DArTseq method reduces genome complexity through digestion with restriction enzymes followed by sequencing of short reads. The selection of appropriate combinations of restriction enzymes enables the isolation of highly informative, low-copy genomic fragments. Up to 90% of DArTseq markers correspond to unique genomic sequences [17,18]. DArTseq analysis generates two datasets: one containing dominant markers and the other comprising codominant markers with identified single-nucleotide polymorphisms (SNPs). At least three times more dominant markers are obtained compared with conventional DArT methods [19]. The DArT technology has also proven to be an efficient diagnostic tool for assessing genotypic diversity [20]. DArT markers have been successfully used to investigate genetic diversity and population structure not only in maize but also in Chinese bread wheat (*Triticum aestivum* L.) [21], oats (*Avena* sp.) [22], pigeon pea (*Cajanus cajan* (L.) Millsp.) [23]. To date, relatively few scientific reports have addressed the application of DArTseq technology to the analysis of Polish maize breeding materials.

These technologies enable the identification of genomic regions associated with various phenotypic traits, including yield, which is fundamental for understanding and manipulating these regions. The extensive genotypic data generated by NGS can be utilized for association mapping. Genome-wide association studies (GWAS) have thus become a powerful methodology for investigating genetic variation and identifying relationships between traits and underlying genetic variation through the exploitation of historical recombination events [24]. Association mapping involves identifying genotype–phenotype correlations in unrelated individuals using specialized statistical methods [25,26,27]. This approach provides opportunities to generate high-quality markers for marker-assisted selection (MAS) [28]. Consequently, association mapping has become a promising approach compared with traditional mapping methods.

Two main types of association mapping are distinguished: genome-wide association mapping (GWAM) and candidate gene association mapping (CGAM). GWAM examines genetic variation across the entire genome to detect association signals for complex traits, whereas CGAM correlates DNA polymorphisms in selected candidate genes with traits of interest [26,27]. Numerous successful applications of association analysis have been reported in cereals, particularly in maize. Recently, GWAM has become a powerful tool for analyzing the genetic architecture of complex traits in various crop species [28,29]. Early association mapping studies in maize did not account for population structure [30]; however, this limitation was addressed by Jonathan K. Pritchard in 2001, who incorporated population structure into maize studies [31]. For several years, maize breeding worldwide has been supported by the application of molecular markers, which has significantly contributed to yield increases not only in the United States but also in other countries, offering substantial potential for enhancing both productivity and the value of maize germplasm [32,33]. Maize, similarly to barley and rice, is one of the best genetically characterized cereal species. Its genome comprises over 32,000 genes distributed across ten chromosomes, with a total size of approximately 2.3 Gbp. A characteristic feature of the maize genome is its high level of polymorphism. Many loci harbor multiple active alleles, and approximately 58% of the genome consists of duplicated DNA sequences, a significant proportion of which are retrotransposons and transposons. Gene regions account for only about 7.5% of the entire maize genome [34].

Over the past two decades, numerous researchers [35,36] have applied molecular biology methods to detect and localize loci determining grain yield and yield-related traits in maize. Since marker-assisted selection enables significant savings in both time and cost, there is a continuous search for new markers linked to yield and its components. Studies conducted by various authors indicate that quantitative trait loci (QTLs) associated with grain yield and its components are distributed across the entire genome. Prasanna et al. [37] demonstrated that the majority of yield-related QTLs in maize are located on chromosomes 1S, 1L, 2S, 5S, 6L, and 8L. Similar results were reported by Beavis et al. [38], who also analyzed test weight and identified QTL regions on chromosomes 1S, 2S, 3S, and 5S. In extensive studies, Ribaut et al. [39] and Veldboom and Lee [40] showed that QTLs associated with kernel row number are located on chromosomes 1L, 4L, 5L, and 9S, whereas QTLs associated with ear number per plant are found on chromosomes 1S, 1L, 3S, 3L, 6L, and 8L. Ribaut et al. [39] also identified QTLs for ear length (chromosomes 1S, 1L, 3S, 3L, 5S, 6L, 8L) and ear diameter (chromosomes 1L, 2L, 4L, 7L, 8L). Additional studies on QTL identification for yield structure traits were conducted by Austin et al. [41] and Melchinger et al. [42]. In contemporary maize breeding, in addition to marker-assisted selection, genomic selection (GS) is increasingly being applied. Genomic selection was first described by Theo Meuwissen et al. [43], who employed statistical models and bioinformatics approaches combined with comprehensive knowledge of plant genomes. One of the key components of GS is highly precise phenotyping of breeding materials, such as maize lines. The term GEBV (Genomic Estimated Breeding Value) refers to the genomic prediction of breeding value [44]. The higher the value of this parameter, the more desirable the material or component for crossing [45].

Plant materials are classified into heterotic groups based on pedigree, diallel crosses, and geographic origin, although these methods have limitations. Consequently, molecular markers are increasingly used to assess genetic similarity between parental lines [20,23]. Advances in plant biotechnology, especially omics technologies and molecular tools, have enhanced crop improvement and agricultural development. This study aimed to identify SilicoDArT and SNP markers associated with candidate genes influencing maize yield using next-generation sequencing, association mapping, and physical mapping.

## 2. Results

**Figure 1:**
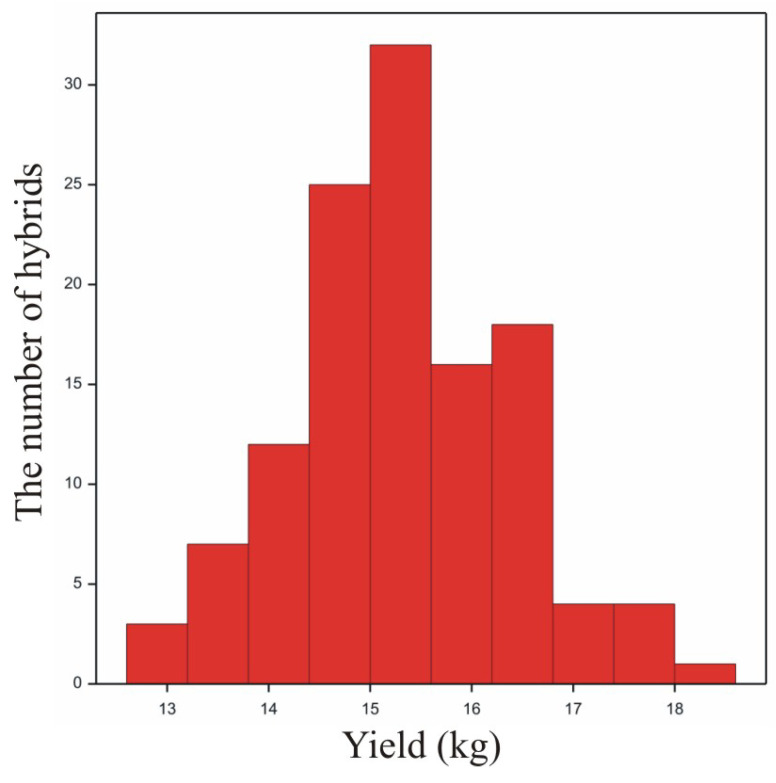
Histogram of the distribution of mean values of 122 maize hybrids.

**Figure 2:**
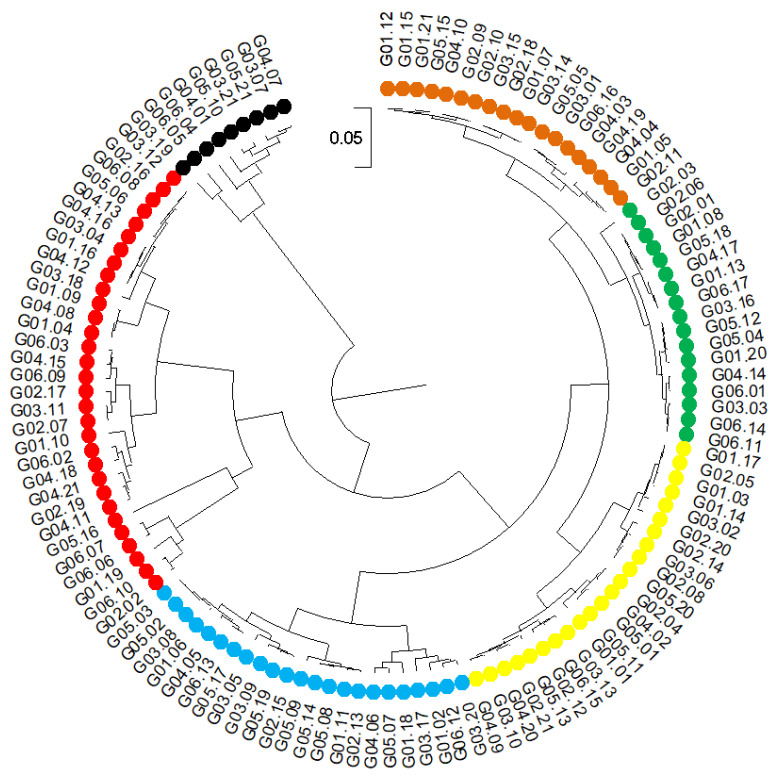
Dendrogram of phenotypic similarity of 122 maize hybrids constructed based on average yields. The optimal tree with the sum of branch lengths = 1538 is shown. Individual colors correspond to six groups of phenotypic similarity of hybrids determined on the basis of yield.

The empirical distribution of mean grain yield values of maize hybrids conformed to a normal distribution (Figure 1). The skewness coefficient of the empirical distribution was 0.1736, while the kurtosis coefficient was—0.1303 (both parameters were not statistically significant). The results of the analysis of variance indicated statistically significant differences among maize hybrids in terms of yield (*p* < 0.001). Mean yield values ranged from 12.67 kg/10 m^2^ (for hybrid G03.19) to 18.52 kg/10 m^2^ (for hybrid G05.16), with an overall mean of 15.28 kg/10 m^2^. The coefficient of variation calculated based on mean values was 7.06%. Phenotypic similarity among hybrids, calculated using Equation (1), ranged from 0 (for hybrids G03.19 and G05.16) to 1 (for seven pairs of hybrids: G03.01 and G05.05, G01.17 and G02.05, G03.16 and G05.12, G01.13 and G06.17, G02.16 and G03.12, G06.11 and G06.14, and G05.17 and G06.13), with an average value of 0.792. The dendrogram constructed based on mean yields allowed the identification of six phenotypic similarity groups comprising 9, 31, 23, 23, 17, and 19 hybrids, respectively (Figure 2).

**Figure 3:**
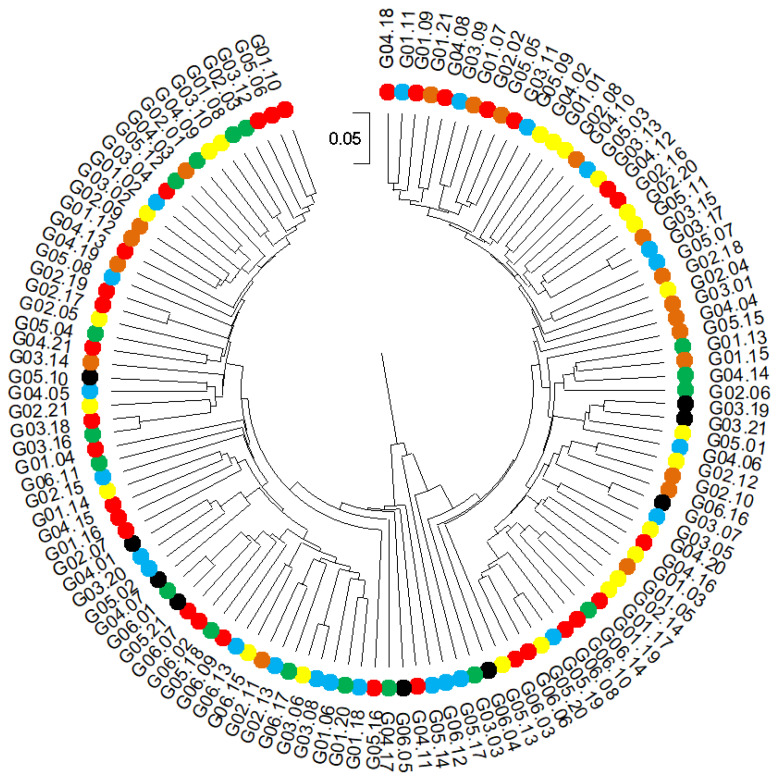
Dendrogram of genetic similarity of 122 maize hybrids constructed based on observations of 25,078 molecular markers. The optimal tree with the sum of branch lengths = 11,560 is shown. Individual colors correspond to six groups of hybrid phenotypic similarity determined based on yield (see Figure 2).

Illumina sequencing resulted in the identification of 60,436 SilicoDArT markers and 32,178 SNP markers (a total of 92,614 molecular markers). A subset of 25,078 markers (18,878 SilicoDArT and 6200 SNP) was used for association mapping. The lowest genetic similarity between hybrids, calculated using Equation (2), was 0.434 (between hybrids G02.09 and G05.17, and G05.14 and G05.17), whereas the highest similarity was 0.957 (between G03.15 and G03.17). The average genetic similarity across all hybrid pairs was 0.659. Based on the identified SilicoDArT and SNP markers, a dendrogram of genetic similarity among the 122 analyzed hybrids was constructed (Figure 3). Three major similarity groups were distinguished: Group I comprising 56 hybrids, Group II comprising 10 hybrids, and Group III comprising 56 hybrids. A comparison of the phenotypic and genotypic dendrograms (Figure 2 and Figure 3) indicates that genetically similar genotypes do not necessarily exhibit similarity in yield performance.

**Figure 4:**
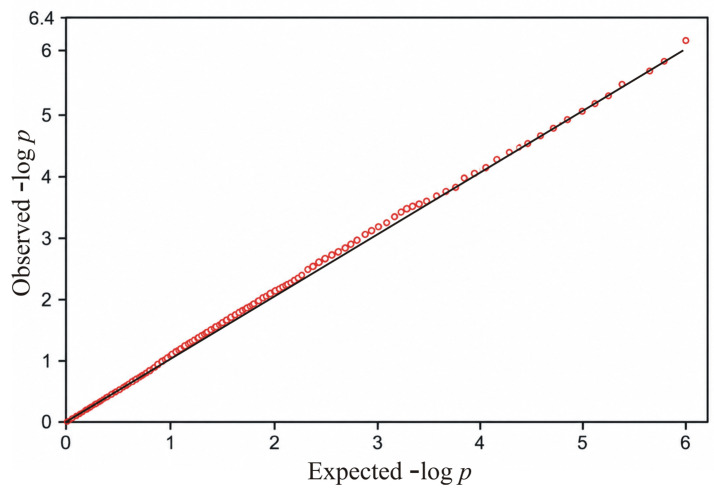
Q-Q plot for assessing model validity.

**Figure 5:**
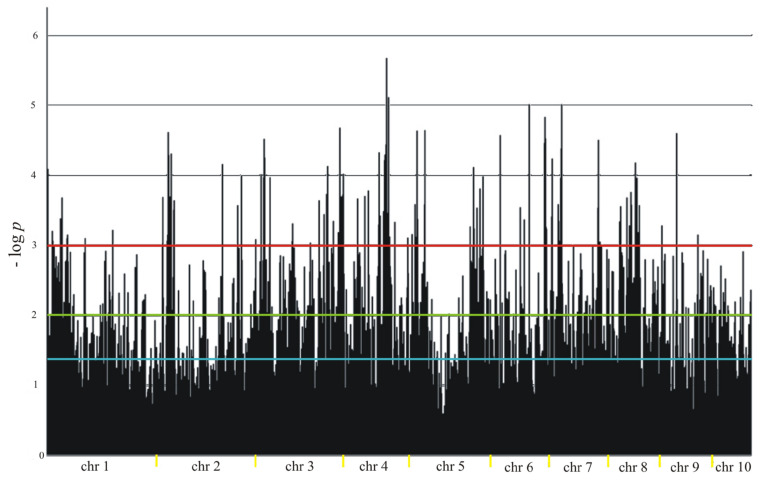
Manhattan plot characterizing the results of genome-wide association mapping of maize; horizontal blue, green, and red lines indicate *p*-values of 0.05, 0.01, and 0.001, respectively. chr—chromosome.

The Manhattan and Q-Q plots were evaluated for evidence of *p* value inflation. The MLM approach substantially reduced false positives, as shown in the Q-Q plot (Figure 4). Association mapping enabled the identification of 2478 markers (1815 SilicoDArT and 663 SNP) that were statistically significant at the 0.05 level and associated with maize yield (Figure 5). Individual markers explained from 2.40% (for 119 markers) to 18.70% (for SNP marker 4772360|F|0-35:C>A-35:C>A) of the total variation in hybrid yield. The number of yield-associated markers varied across chromosomes, with counts of 376, 191, 432, 296, 284, 188, 262, 241, 99, and 109, respectively (Figure 5). Among the identified markers, 47 were highly statistically significant (Logarithm of the Odds − LOD > 4.0) (Table 1, Figure 5). Of these, 33 were SilicoDArT markers, and 14 were SNP markers. Their chromosomal distribution was highly uneven, ranging from one marker (on chromosomes 8 and 9) to twelve markers (on chromosome 6) (Table 1). No highly significant yield-associated markers were identified on chromosome 10. The proportion of yield variation explained by the selected markers ranged from 11.20% (for two SilicoDArT markers: 4767251 and 4770317) to 18.70% (for SNP marker 4772360|F|0-35:C>A-35:C>A) (Table 1).

For further stages of the breeding program, ten markers were proposed that explain a substantial proportion of yield variability, ranging from 13.30% to 18.70% (Table 2). Two SilicoDArT markers (4593047 and 9693261), located on chromosomes 7 and 3, respectively, are particularly noteworthy because they are located within genes that potentially influence maize yield. Marker SilicoDArT 24028032, located near (809 bp) the gene encoding long-chain acyl-CoA synthetase (LACS) on chromosome 4, may also play an important role. This gene is involved in the conversion of free FAs into fatty acyl-CoA thioesters, which play crucial roles in FA catabolism, lipid synthesis, and storage [46]. Additionally, all LACSs genes showed elevated expression levels during seed development at 2, 4, and 8 DAP, suggesting their potential role in early FA accumulation in maize seeds [47]. Table 3 presents primers designed to identify molecular markers significantly associated with yield. These markers can be used to select high-yielding genotypes in breeding programs.

The Gene Ontology (GO) analysis of candidate genes identified in proximity to significant SNP and SilicoDArT markers revealed that the associated loci are involved in several key biological pathways related to plant metabolism, stress response, signaling, and cellular organization. The predominant biological processes represented among the identified candidate genes were lipid metabolism and regulation of gene expression/signaling pathways. Genes such as *long-chain acyl-CoA synthetase* (*LACS*) and *sphinganine C4-monooxygenase* were associated with lipid biosynthesis and membrane remodeling processes, suggesting the involvement of membrane-associated stress adaptation mechanisms. Lipid metabolism plays a crucial role in maintaining membrane integrity and regulating signaling processes. Another highly represented category included genes involved in transcriptional regulation and intracellular signaling, particularly transcription factor MYB60 and rho GDP-dissociation inhibitor 1. These proteins are known to participate in stress-responsive regulatory networks, including responses to drought, hormonal signaling, and cellular communication pathways. Additional biological processes identified in the analysis included water transport, carbohydrate metabolism, proteolysis, hormonal regulation, and RNA processing. The presence of the *aquaporin NIP2-1-like* gene suggests potential involvement in water homeostasis and abiotic stress adaptation, whereas *starch synthase homolog 1* indicates participation in carbohydrate accumulation and energy metabolism. Furthermore, *adenylate isopentenyl-transferase*, associated with *cytokinin biosynthesis*, points to the contribution of hormonal regulation to the analyzed trait. Genes involved in protein turnover and RNA maturation, including *aspartic proteinase oryzasin-1* and *CRM-domain* containing factor *CFM3*, further support the complex molecular background of the studied loci. The Molecular Function analysis demonstrated a clear predominance of genes with catalytic activity. Enzymatic functions were mainly represented by proteins involved in lipid metabolism, starch biosynthesis, and cytokinin synthesis, indicating intensive metabolic activity within the identified genomic regions. Transferase activity also constituted an important functional category, reflecting the involvement of biosynthetic pathways related to carbohydrate and hormone metabolism. In addition, transporter activity was represented by *aquaporin NIP2-1-like*, highlighting the significance of membrane transport processes. The presence of transcription factor activity, represented by *MYB60*, confirms the importance of transcriptional regulation in controlling the analyzed phenotype. Other identified molecular functions included protease activity and GDP-dissociation inhibitor activity, suggesting contributions from protein degradation and signal transduction pathways. The Cellular Component classification indicated that most candidate gene products are localized within cellular membranes and energy-related organelles. The plasma membrane represented one of the most enriched cellular compartments, primarily due to the presence of aquaporins and lipid metabolism-related proteins. This observation supports the hypothesis that membrane transport and membrane-associated signaling processes play a major role in the biological mechanisms underlying the studied trait. A substantial proportion of genes were also associated with chloroplasts and plastids, including *starch synthase* and *CFM3*, suggesting links to photosynthesis, carbon metabolism, and organellar gene regulation. *MYB60* was localized to the nucleus, consistent with its role as a transcriptional regulator, whereas *rho GDP-dissociation inhibitor 1* was associated with the cytoplasm and intracellular signaling processes. Additional localization categories included the endomembrane system, mitochondria, and vacuolar/apoplastic compartments, further emphasizing the complexity of cellular processes associated with the identified loci. Overall, the GO analysis indicates that the genomic regions associated with the analyzed markers participate in interconnected regulatory and metabolic networks related to stress adaptation, membrane function, hormonal signaling, transcriptional regulation, and energy metabolism. The coexistence of genes involved in water transport, lipid remodeling, carbohydrate metabolism, and signal transduction suggests that the studied trait is likely controlled by a complex polygenic architecture involving coordinated physiological and molecular responses.

## 3. Discussion

Since the mid-1990s, numerous research centers worldwide have conducted intensive studies on the structure and function of the maize genome using advanced biotechnological and molecular biology methods. As a result of comprehensive breeding experiments, phenotypic observations, and genetic analyses, numerous quantitative trait loci (QTLs) associated with important quantitative traits, such as yield and yield components, have been identified. Moreover, heterosis breeding in maize consistently aims to exploit hybrid vigor, shorten the breeding process (e.g., through the use of doubled haploid lines), and improve quality while reducing the costs associated with seed production. A key priority for breeders remains the development of high-yielding and disease-resistant maize varieties [48].

In the present study, 122 maize genotypes were analyzed at both phenotypic and genotypic levels in order to identify molecular markers linked to genes determining yield. Based on phenotypic observations (yield performance), an analysis of variance revealed statistically significant differences among maize hybrids (*p* < 0.001). The empirical distribution of mean yield values followed a normal distribution (Figure 1), with a skewness coefficient of 0.1736 and a kurtosis coefficient of 0.1303.

However, phenotypic analysis alone is insufficient for the effective selection of parental components for heterotic crosses, as traditional breeding methods are increasingly inadequate in the context of rapid technological progress. In response to this challenge, modern breeding programs employ high-throughput genomic techniques to improve new crop varieties, including maize [49]. A genomics-oriented approach enables the identification of coding regions, providing insight into gene structure and protein function, as well as intergenic regions [50]. Advances in high-throughput DNA sequencing technologies, enabling the analysis of entire genomes and transcriptomes, have significantly enhanced research capabilities in many plant species, including maize [51]. The introduction of next-generation sequencing (NGS) has facilitated the analysis of species beyond classical model organisms with small genomes, such as *Arabidopsis thaliana* (L.) Heynh. Current research focuses primarily on economically important crops, including cereals, coffee, maize, and sugarcane [52]. In 2024, the genomes of 500 plant species were published, including 370 sequenced for the first time. Tracking and sharing published plant genomes (currently covering over 1800 species) is an invaluable service for plant researchers [53,54].

These advances have enabled the detection of SNPs and their association with specific traits, as well as the development of genomic selection approaches that allow monitoring of entire genome segments in recombination breeding programs. It is essential that practical breeding objectives are supported by comprehensive fundamental research that contributes to the advancement of knowledge in plant genetics, physiology, and biochemistry. In recent years, numerous studies have focused on identifying molecular markers functionally associated with important traits in maize. For example, Bocianowski et al. [55] applied NGS technology and association mapping to identify markers related to heterosis effects in maize, while Sobiech et al. [56] identified markers associated with resistance to *Fusarium* spp. infections. NGS technologies are widely used for genome and transcriptome sequencing, studying protein–DNA/RNA interactions, assessing DNA methylation levels, discovering new DNA polymorphisms, and conducting metagenomic studies [57]. These technologies enable the simultaneous analysis of multiple DNA fragments, library preparation, and the generation of gigabases of genomic data in a single sequencing run [58,59].

This significantly increases not only the number of analyzed samples but also the reliability of sequencing results, which is particularly important when genetic differences between genotypes are small [49]. Moreover, the cost and time required for sequencing per unit of information are substantially lower compared to traditional capillary sequencing methods [60]. cDNA sequence analysis provides information on actively transcribed sequences in specific tissues or organs and, despite certain limitations, remains highly valuable for breeders [49,61,62]. NGS technologies also enable both qualitative and quantitative analyses of gene expression under various conditions, and the results of these analyses are increasingly used in association mapping studies [63,64,65,66]. In the present study, next-generation sequencing identified 60,436 SilicoDArT markers and 32,178 SNP markers. A total of 25,078 markers were used for association mapping. Based on these markers, genetic similarity among the analyzed genotypes was estimated, ranging from 0.434 (between hybrids G02.09 and G05.17, and G05.14 and G05.17) to 0.957 (between G03.15 and G03.17) (Figure 3). A genetic similarity dendrogram constructed from SilicoDArT and SNP markers revealed three distinct groups (Figure 3). Notably, genetic grouping did not correspond to phenotypic grouping based on yield performance (Figure 2 and Figure 3), which is consistent with findings reported by Kozak et al. [67]. The fact that genetic grouping is not the same as phenotypic grouping is crucial information for breeders, including maize breeders. It means that when selecting materials, breeders must consider both types of variability: phenotypic and genetic. Selecting markers to identify candidate genes related to maize yield should facilitate the selection process. It certainly shortens it. Hybrid yield can also be determined based on traits other than yield. These can include kernel number, kernel size, and moisture content, or lack thereof. However, the most important trait for breeders is seed yield, which is why this trait is given the greatest importance.

Association mapping identified 2478 markers (1815 SilicoDArT and 663 SNP) significantly associated with maize yield at the 0.05 level (Figure 5). Individual markers explained between 2.4% and 18.7% of the total variation in hybrid yield. Among these, 47 markers were highly statistically significant (LOD > 4.0) (Table 1). For further stages of the breeding program, ten markers were selected that explain a substantial proportion of yield variability, ranging from 13.30% to 18.70%. These markers may be effectively applied in the selection of high-yielding maize genotypes (Table 2 and Table 3). One of the most interesting associations identified in this study involved the SilicoDArT marker (Clone ID 24028032), located approximately 809 bp from a putative *long-chain acyl-CoA synthetase* (*LACS*)-related sequence. However, it should be emphasized that the present study identifies statistical marker–trait associations rather than direct causal relationships. Therefore, the detected association may reflect linkage disequilibrium with genes involved in physiological processes affecting yield. Literature reports indicate that this gene may influence maize yield. *LACSs*, which convert free FAs into fatty acyl-CoA thioesters, play crucial roles in FA catabolism, lipid synthesis and storage, epidermal wax synthesis, and stress tolerance [46,68,69]. In *Arabidopsis*, more than three *LACS* proteins have been shown to be involved in keratin and epidermal wax production. Furthermore, the biochemical homeostasis of epidermal wax in *Arabidopsis* can enhance plant tolerance to water deficiency, salt stress, drought stress, and ABA treatment [70,71,72]. These studies strongly suggest that LACS proteins play a vital role in responding to stress via regulating wax biosynthesis. Nevertheless, because *Arabidopsis* and maize differ substantially in their developmental and physiological characteristics, these findings should be treated only as indirect functional indications rather than direct evidence for maize yield regulation. In maize research, the levels across diverse tissues demonstrated that the majority of LACS genes exhibited the highest expression in the meiotic tassel. The high demand for LACS activities in floral tissues is linked to the strong expression of other lipid metabolic genes in flowers, indicating their involvement in active FA metabolism during meiotic tassel development. Additionally, all LACS genes showed elevated expression levels during seed development at suggesting their potential role in early FA accumulation in maize seeds [73,74]. Such processes may indirectly contribute to grain formation and yield stability under environmental stress conditions. However, additional transcriptomic, functional, and validation studies are necessary to determine the biological significance of this genomic region in maize yield formation. Importantly, the lack of complete agreement between genetic grouping and phenotypic performance observed in this study confirms the complex quantitative nature of maize yield. Molecular similarity among genotypes does not necessarily correspond to similar agronomic performance because yield is strongly influenced by polygenic inheritance.

## 4. Materials and Methods

### 4.1. Plant Material

The plant material used in this study was obtained from Plant Breeding Smolice Ltd. (Kobylin, Poland), belonging to the Plant Breeding and Acclimatization Institute-National Research Institute Group (51°42′58.904″ N, 17°13′29.13″ E). The experimental material consisted of 122 maize hybrids, designated as follows: G01.01-G01.21; G02.01-G02.21; G03.01-G03.21; G04.01-G04.21; G05.01-G05.21; G06.01-G06.17. The hybrids were developed through crosses between inbred lines of diverse genetic origin, which were comprehensively characterized by Sobiech et al. [75].

### 4.2. Field Experiments

The experiment was conducted in 2022 on plots with an area of 10 m^2^, arranged in a randomized complete block design with three replications, at Małopolska Plant Breeding Kobierzyce Ltd. (Kobierzyce, Poland; 50°58′019.411″ N, 16°55′047.323″ E). Yield assessment was performed on ten plants sampled from each plot for each replicate. The total number of plants measured for each hybrid was 30.

Maize is a plant with a long growing season, typically lasting 140 to 180 days in Polish climatic conditions. Maize was sown on 25 April (when the soil temperature at a depth of 5–10 cm reached 10 °C). Harvest occurred on 15 October (at full maturity, with grain moisture below 30%). The sowing density was 80,000 plants ha^−1^. The sowing depth was 5 cm. Regarding fertilization, maize has very high nutrient requirements. Nitrogen (150 kg h^−1^) was applied in two doses: pre-sowing and top-dressing (up to the 4–6 leaf stage). Phosphorus was applied at 80 kg ha^−1^, and potassium at 150 kg ha^−1^. In terms of irrigation and water conditions, maize manages water efficiently (low transpiration rate), but due to its enormous biomass, it requires significant amounts of water during critical phases: flowering and intensive growth (July–August). Sprinkler irrigation was used as needed, maintaining soil moisture at 60–80% of field water capacity.

### 4.3. Weather Conditions

**Figure 6:**
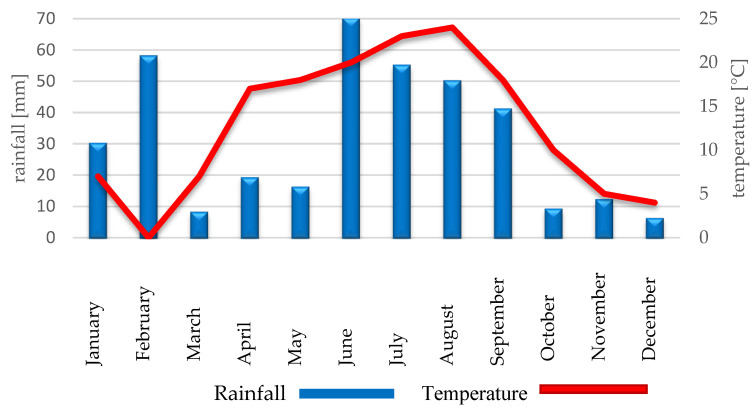
Average monthly temperature and average monthly precipitation in Kobierzyce in 2022.

The 2022 growing season was favorable for maize growth and development in terms of meteorological conditions. May was a very warm and relatively humid month, with an average monthly temperature of 15.9 °C and monthly precipitation of approximately 50.2 mm. Low humidity and a lack of significant rainfall contributed to a deepening drought. June, July, and August were characterized by above-normal average temperatures of 24.9 °C, 22.8 °C, and 19.3 °C, respectively. In contrast to May, June (55.8 mm) and July (60.2 mm) had higher rainfall totals during this period, which favorably affected plant growth. September was a warm month (16.7 °C) with low precipitation (39.5 mm), as was October (9.2 °C, 26.6 mm). The relatively dry and warm weather did not favor the development of fungal diseases throughout the maize growing season. The meteorological conditions in Kobierzyce are shown in Figure 6.

### 4.4. DNA Isolation

Genomic DNA was isolated from 122 maize hybrids using a commercial reagent kit provided by Symbios, Chassieu, France. The extracted DNA samples were subsequently subjected to next-generation sequencing. DNA concentration and purity were determined using a DS-11 spectrophotometer (DeNovix, Wilmington, DE, USA). The isolated DNA was normalized to a uniform concentration of 100 ng/μL by dilution with deionized distilled water.

### 4.5. Genotyping

Genotyping was performed using DArTseq technology, based on next-generation sequencing. DNA samples isolated from 122 maize hybrids, each with a volume of 50 μL and a concentration of 100 ng/μL, were transferred onto two 96-well Eppendorf plates for the identification of SilicoDArT and SNPs. The analyses were conducted by Diversity Arrays Technology at the University of Canberra, Bruce, Australia. The applied methodologies are described in detail on the Diversity Arrays Technology website (https://www.diversityarrays.com/technology-and-resources/dartseq/, accessed 15 January 2024).

### 4.6. Statistical Analysis and Association Mapping

The conformity of the empirical distribution of maize yield data to the normal distribution was assessed using the Shapiro-Wilk test [76]. Analysis of variance (ANOVA) was performed to determine the significance of the genotype effect on yield. Phenotypic similarity among the 122 maize hybrids was calculated according to the following formula [77]:

(1) $$S_{F,i,j}=1-\frac{\left|{\bar{y}}_{i}-{\bar{y}}_{j}\right|}{max\left({\bar{y}}_{.}\right)},$$

where $S_{F,i,j}$ denotes the phenotypic similarity between hybrid *i* and hybrid *j*, ${\bar{y}}_{i}$ is the mean yield of hybrid *i*, ${\bar{y}}_{j}$ is the mean yield of hybrid *j*, and $max\left({\bar{y}}_{.}\right)$ represents the maximum mean yield among all analyzed hybrids.

Clustering of hybrids based on phenotypic similarity was performed using similarity coefficients calculated according to Equation (1) and presented as a dendrogram constructed using the unweighted pair group method with arithmetic mean (UPGMA) [78].

Genetic similarity among the 122 maize hybrids was estimated based on molecular marker data using Nei’s coefficient [79]:

(2) $$S_{G,i,j}=\frac{2N_{ij}}{N_{i}+N_{j}},$$

where $S_{G,i,j}$ denotes the genetic similarity between hybrid *i* and hybrid *j*, $N_{i}$ is the number of alleles present in hybrid *i*, $N_{j}$ is the number of alleles present in hybrid *j*, and $N_{ij}$ is the number of alleles shared by both hybrids.

Clustering based on genotypic similarity was carried out using similarity coefficients calculated according to Equation (2) and visualized as a dendrogram constructed using the UPGMA method [78]. Evolutionary analyses were performed using MEGA v.12 [80].

Association mapping was conducted based on combined genotypic and phenotypic data using a genome-wide association study (GWAS) analysis. Genotypic data were obtained from DArTseq analysis, while phenotypic data were derived from yield measurements. Only SilicoDArT and SNP sequences meeting the following criteria were included in the association analysis: a single SilicoDArT and/or SNP per sequence (69 nt), minor allele frequency (MAF) > 0.25, and a proportion of missing data < 10%. Association mapping was performed using a mixed linear model (MLM) approach, with population structure estimated through eigenvalue decomposition from principal component analysis (PCA) and modeled as random effects [81,82]. The significance of associations between yield and SilicoDArT and SNP markers was evaluated using *p*-values adjusted for multiple testing with the Benjamini–Hochberg procedure. The Benjamini–Hochberg method is a statistical technique that controls the false discovery rate (FDR) during multiple hypothesis testing. It is less stringent than the Bonferroni correction, offering greater power to detect significant differences while reducing the false positive rate. The Manhattan and Q-Q plots were plotted. All statistical analyses and result visualizations were carried out using Genstat 24.2 [83].

### 4.7. Physical Mapping

SilicoDArT and SNP marker sequences selected based on GWAS were subjected to BLAST (Basic Local Alignment Search Tool, version 2.17.0) analysis, which involves searching databases to identify sequences with high homology to the selected marker sequences. Publicly available web-based genome browsers and databases were used for this purpose, including the CEPH genotype database (http://www.cephb.fr/en/cephdb/, accessed on 10 April 2026), NCBI Map Viewer (https://www.ncbi.nlm.nih.gov/gdv/, accessed 10 April 2026), UCSC Genome Browser (http://genome.ucsc.edu/, accessed on 10 April 2026), and Ensembl Map View (https://www.ensembl.org/index.html, accessed on 10 April 2026). These tools were applied to determine the chromosomal locations of the analyzed sequences.

### 4.8. Functional Analysis of Gene Sequences

Functional analysis was performed using Blast2GO 6.0. All gene sequences located within chromosomal regions identified through BLAST analysis were subjected to further examination. The aim of this analysis was to obtain information on the biological functions of gene sequences located within the defined chromosomal regions.

### 4.9. GO Enrichment Analyses on Candidate Genes

Gene ontology (GO) analyses of the predicted target genes were conducted using GO (https://www.geneontology.org/, accessed on 12 May 2026). An analytical tool from the DAVID bioinformatic tools (https://davidbioinformatics.nih.gov/summary.jsp, accessed on 12 May 2026) was used, which is continuously updated with GO annotations.

## 5. Conclusions

The present study demonstrated significant phenotypic and genotypic variability among the analyzed maize hybrids, confirming the suitability of the examined material for advanced genetic and breeding analyses. The observed statistically significant differences in yield indicate substantial breeding potential and justify further selection efforts. The application of next-generation sequencing (NGS) technologies, combined with DArTseq genotyping, enabled the identification of a large number of SilicoDArT and SNP markers, providing a high-resolution insight into the genetic structure of the analyzed hybrids. Genome-wide association analysis (GWAS) proved to be an effective tool for identifying molecular markers associated with maize yield. A total of 2478 significant markers were detected, including 47 highly significant ones (LOD > 4.0), confirming the complex genetic architecture of yield as a quantitative trait controlled by multiple loci distributed across the genome. Among the identified markers, ten were selected as particularly valuable due to their high contribution to yield variability (13.30–18.70%). These markers represent promising candidates for implementation in marker-assisted selection (MAS) and genomic selection (GS), enabling more efficient identification of high-yielding genotypes. Overall, the integration of phenotypic evaluation with high-throughput genotyping and association mapping provides a powerful approach for accelerating maize breeding programs. The identified marker–trait associations provide a basis for further studies on the genetic architecture of maize yield and may support future breeding applications after functional and multi-environment validation.

## Figures and Tables

**Table 1:** The 47 markers with a highly statistically significant effect (LOD > 4.0) on the yield of maize hybrids.

Chr. ^1^	Marker Type	CloneID ^2^	Effect	Percentage of Explained Variation	LOD ^3^
1	SNP	4772360|F|0-35:C>A-35:C>A	−0.995	18.70	6.40
1	SNP	4765631|F|0-17:T>C-17:T>C	−0.919	16.00	5.52
1	SilicoDArT	4769827	0.789	11.40	4.09
2	SNP	2428319|F|0-9:A>G-9:A>G	−0.91	13.10	4.62
2	SNP	4592045|F|0-15:G>C-15:G>C	0.81	12.10	4.28
2	SNP	4773910|F|0-38:A>C-38:A>C	0.832	12.10	4.30
2	SilicoDArT	2453978	0.758	11.70	4.16
3	SilicoDArT	4767251	−0.742	11.20	4.01
3	SilicoDArT	4581108	−0.756	11.50	4.09
3	SilicoDArT	67861110	−0.83	12.60	4.44
3	SilicoDArT	4590245	0.848	12.80	4.51
3	SilicoDArT	9686149	−1.014	12.00	4.25
3	SilicoDArT	24029540	−0.866	11.60	4.13
3	SilicoDArT	9693261	0.811	13.30	4.67
4	SilicoDArT	4770317	0.967	11.20	4.02
4	SilicoDArT	2461977	−0.784	12.20	4.33
4	SilicoDArT	2447622	0.778	11.90	4.22
4	SilicoDArT	25942471	0.775	12.10	4.29
4	SilicoDArT	4580178	−0.786	12.60	4.44
4	SNP	4584260|F|0-52:G>C-52:G>C	−0.915	16.50	5.67
4	SNP	2529995|F|0-34:C>T-34:C>T	−0.79	11.30	4.05
4	SilicoDArT	2496745	−0.878	15.20	5.28
4	SilicoDArT	24028032	−0.845	14.70	5.11
4	SNP	24028032|F|0-39:A>C-39:A>C	0.853	14.40	5.03
4	SNP	4773974|F|0-56:G>T-56:G>T	−0.777	12.30	4.36
5	SilicoDArT	4775428	−0.805	13.20	4.64
5	SilicoDArT	2472314	−1.026	13.20	4.64
5	SilicoDArT	4774003	−0.766	11.50	4.12
6	SilicoDArT	4585699	-0.764	11.90	4.22
6	SNP	2410784|F|0-59:A>G-59:A>G	−0.797	13.00	4.57
6	SilicoDArT	9681926	1.311	14.40	5.01
6	SNP	2426321|F|0-22:G>T-22:G>T	−0.763	11.90	4.22
6	SilicoDArT	4779010	−0.782	12.50	4.41
6	SilicoDArT	25001017	−0.788	12.60	4.46
6	SilicoDArT	4579008	0.81	12.50	4.42
6	SilicoDArT	2447403	0.807	11.40	4.06
6	SilicoDArT	4585520	0.951	13.80	4.83
6	SilicoDArT	25948407	0.849	11.30	4.06
6	SNP	4778336|F|0-17:A>G-17:A>G	0.852	11.70	4.16
6	SilicoDArT	7049369	0.855	12.80	4.52
7	SilicoDArT	4592089	−0.789	11.90	4.24
7	SNP	4583885|F|0-64:A>G-64:A>G	0.789	12.40	4.38
7	SilicoDArT	4593047	−0.84	14.40	5.01
7	SilicoDArT	4582042	−0.769	11.80	4.19
7	SilicoDArT	4767434	0.841	12.80	4.50
8	SilicoDArT	4593805	0.76	11.70	4.18
9	SNP	9710633|F|0-55:T>C-55:T>C	−0.799	13.10	4.60

^1^ Chr.—chromosome, ^2^ ID—unique identifier, ^3^ LOD—Logarithm of the Odds.

**Table 2:** Location of specific markers in the maize genome.

Marker Type	CloneID ^1^	Chr. ^2^	Marker Location	Genome Location
SNP	4772360|F|0-35:C>A-35:C>A	1	1248434	21,629 bp at 5′ side: uncharacterized protein loc100279889 234,840 bp at 3′ side: uncharacterized protein isoform x1
SNP	4584260|F|0-52:G>C-52:G>C	4	186981952	58,601 bp at 5′ side: uncharacterized protein loc100501967 30,041 bp at 3′ side: sphinganine c4-monooxygenase 1 isoform x1
SNP	4765631|F|0-17:T>C-17:T>C	1	1248585	21,780 bp at 5′ side: uncharacterized protein loc100279889 234,689 bp at 3′ side: uncharacterized protein isoform x1
SilicoDArT	2496745	4	187167577	3369 bp at 5′ side: aquaporin nip2-1-like 48,077 bp at 3′ side: starch synthase homolog 1
SilicoDArT	24028032	4	191094591	41,711 bp at 5′ side: transcription factor myb60 809 bp at 3’ side: long chain acyl-coa synthetase (LACSs)
SNP	24028032|F|0-39:A>C-39:A>C	4	191094591	41,711 bp at 5′ side: transcription factor myb60 809 bp at 3′ side: long chain acyl-coa synthetase (LACSs)
SilicoDArT	4593047	7	32856843	uncharacterized protein isoform x1 uncharacterized protein loc100193457
SilicoDArT	9681926	6	153046544	5011 bp at 5′ side: aspartic proteinase oryzasin-1 1524 bp at 3′ side: rho gdp-dissociation inhibitor 1
SilicoDArT	4585520	6	174290081	22,169 bp at 5′ side: adenylate isopentenyltransferase 4684 bp at 3′ side: crm-domain containing factor cfm3
SilicoDArT	9693261	3	232772327	uncharacterized protein loc100276737

^1^ ID—unique identifier, ^2^ Chr.—chromosome.

**Table 3:** Primers for identifying specific SilicoDArT and SNP markers.

Marker Type	CloneID ^1^	Temp. (°C)	Product Length (bp)	Sequences (5′-3′)
SNP	4772360|F|0-35:C>A-35:C>A	58–60	188	F:CATGTCCATTTCCTCTGCAG R:TGAGACCCTCATTCGCTCTG
SNP	4584260|F|0-52:G>C-52:G>C	58–60	349	F:CCGGGATATATACCCCTGCA R:TACTCGCCCATGCAAGTCTG
SNP	4765631|F|0-17:T>C-17:T>C	59–60	385	F:GAGCATTCTCCATGCTGCAG R:GGCCTCATTGACGTGAGGAA
SilicoDArT	2496745	59–62	419	F:GCTTGCTCTTGTGCCTGCAG R:CCCTGGTCGTAAGCCAGTTT
SilicoDArT	24028032	56–59	158	F:GACTCGATGTTGTTCTGCAG R:CCAGTGCCTTCTCCCATGAA
SNP	24028032|F|0-39:A>C-39:A>C	56–59	158	F:GACTCGATGTTGTTCTGCAG R:CCAGTGCCTTCTCCCATGAA
SilicoDArT	4593047	59–60	223	F:TAGGTCTAATGATCGTAAAACTGCAG R:TGAAACTGTCAGTGCAGCCA
SilicoDArT	9681926	60–62	206	F:TGCACCATTGCCAACTGCAG R:GGATGGGGAAGAGGGGTACT
SilicoDArT	4585520	55–57	388	F:TACAACCATAGCTTCTGCAG R:CACGGTGGTATATAGGGGGT
SilicoDArT	9693261	58–60	249	F:GCATTTGATTGAAACATTCTGCAG R:TCATCGGGCTTGCTTACGTT

^1^ ID—unique identifier.

## Data Availability

The datasets generated and analyzed during the current study are available from the corresponding author on reasonable request.

## Abbreviations

The following abbreviations are used in this manuscript:

NGS	Next-generation sequencing
GWAS	Genome-wide association studies
LOD	Logarithm of the Odds
MAS	Marker-assisted selection
GS	Genomic selection
RAD	Restriction site Associated DNA
MSG	Multiplexed Shotgun Genotyping
GBS	Genotyping-by-sequencing
GWAM	Genome-wide association mapping
CGAM	Candidate gene association mapping
QTL	Quantitative trait locus
BLUP	Best Linear Unbiased Prediction
RR-BLUP	Ridge Regression BLUP
GBLUP	Genomic BLUP
SNP	Single-nucleotide polymorphism
ANOVA	Analysis of variance
UPGMA	Unweighted pair group method with arithmetic mean
MAF	Minor allele frequency
MLM	Mixed linear model
PCA	Principal component analysis
BLAST	Basic Local Alignment Search Tool
